# Retinal Nerve Fiber Layer Thickness and Oxidative Stress Parameters in Migraine Patients without Aura: A Pilot Study

**DOI:** 10.3390/antiox9060494

**Published:** 2020-06-05

**Authors:** Adriana Elena Bulboacă, Ioana C. Stănescu, Sorana D. Bolboacă, Angelo C. Bulboacă, Gyorgy I. Bodizs, Cristina A. Nicula

**Affiliations:** 1 Department of Pathophysiology, Iuliu Haţieganu University of Medicine and Pharmacy, 400012 Cluj-Napoca, Romania; adriana_bulboaca@yahoo.com; 2 Department of Neurology and Pediatric Neurology, Iuliu Haţieganu University of Medicine and Pharmacy, 400012 Cluj-Napoca, Romania; angelo.bulboaca@yahoo.com; 3 Department of Medical Informatics and Biostatistics, Iuliu Haţieganu University of Medicine and Pharmacy, 400349 Cluj-Napoca, Romania; 4 Clinical Rehabilitation Hospital, 400437 Cluj-Napoca, Romania; gbodizs@yahoo.com; 5 Department of Ophthalmology, Iuliu Haţieganu University of Medicine and Pharmacy, 400006 Cluj-Napoca, Romania; niculacristina65@yahoo.com

**Keywords:** retinal nerve fiber layer (RNFL) thickness, migraine, optical coherence tomography (OCT), oxidative stress

## Abstract

Background: Migraine is one of the most common disorders and its pathophysiological mechanisms are still under research, oxidative stress being emphasized as an important contributor. This study aimed to analyze the retinal nerve fiber layer (RNFL) thickness and oxidative/anti-oxidant balance in migraine patients. Methods: Two groups of subjects were evaluated: a group of patients with migraine and a control group of healthy volunteers. RNFL thickness was assessed for all subjects by the ocular coherence tomography spectral domain (OCT-SD). The oxidative stress parameter, namely nitric oxide (NOx), malondialdehyde (MDA), and total oxidative stress (TOS) were assessed. The antioxidant capacity of plasma was evaluated by assessing the level of catalase, and total anti-oxidative (TOS) capacity. Migraine severity was graded using the Migraine Disability Assessment Score (MIDAS) questionnaire. Results: All the oxidative stress parameters (NOx, MDA, and TOS) were significantly increased, and both parameters for anti-oxidative status were significantly decreased in the migraine group compared with the control group (*p* < 0.0001). Significant correlations with all the quadrants and different oxidative stress parameters were found, most involved being temporal quadrant. A significant positive correlation between catalase and macular RNFL thickness (inner ring, temporal quadrant) in migraine patients, for both eyes, was observed (*p* = 0.014 for the right eye and *p* = 0.12 for the left eye). Conclusion: The assessment of the oxidative stress/anti-oxidative balance together with RFLN thickness can constitute a promising method to evaluate the progression of the diseases. It can also contribute to the estimation of the efficiency of various therapies targeting oxidative stress and associated inflammation.

## 1. Introduction

Migraine is a common chronic disorder characterized by an episodic headache accompanied by various neurological, gastrointestinal and/or autonomic disturbances. A relevant percent of the population (up to 21% of women and 6% of men) suffer from migraine attacks associated with activity impairment and disability [1]. According to the World Health Organization, headache disorders constitute one of the ten most disabling conditions for both genders, while, for women, they rank among the five most disabling states [1]. Approximately 30% of migraine patients experience transient, fully reversible, neurological, or visual symptoms (aura) preceding the attack [2,3]. In both types of migraine patients, with or without aura, there is more extensive responsiveness to light stimulation in the visual cortex [4,5]. During attacks, the activity of the visual cortex is increased as a response to increasing intensities of light stimulation [6]. Different pathophysiological mechanisms had been proposed to explain the migraine, but the issue is still far from being fully clarified. One of the most relevant pathophysiological mechanisms of migraine is the increase in oxidative stress [7]. The neurovascular system also remains one of the most important mechanisms involved in the pathogenesis of migraine, and, consecutive, hypo-perfusion might involve other areas besides the brain, such as the retina, leading to axonal loss and changing of the retinal nerve fiber layers thickening [8,9,10]. Hemodynamic changes (characterized by hypo-perfusion), hypercoagulability status and altered endothelial functions are between pathophysiological mechanisms that are suggested to be associated with migraine [11,12]. Experimental and clinical studies showed that inflammation triggered and amplified by oxidative stress could constitute other mechanisms associated with migraine attacks [13,14,15]. Oxidative stress enhancement during migraine attacks can arise from different mechanisms, including a high rate of energy production by the mitochondria, calcium overload into the cells, neuronal excitotoxicity, neuroinflammation, the activation of microglia, or the activation of neuronal nicotinamide adenine dinucleotide phosphate (NADPH) oxidase [7]. Proinflammatory cytokines such as IL-1β, IL-6, and TNF-α proved to have high concentrations in serum in patients with migraine, indicating that inflammation plays a significant role in migraine pathogenesis [16]. During a migraine attack, sensitization of the trigeminal vascular system (TGVS), including the network of intra- and extracranial meningeal blood vessels and ocular structures, affecting the vascular tone and the transmission of pain signals, is observed [17,18,19]. The hypo-perfusion of the eyes’ structures occurring during a migraine attack (due to cerebral vessels vasoconstrictions) and repetitive attacks can lead to permanent damage of the brain and eye structures, including retina [20,21].

Since its introduction in the clinical practice, optical coherence tomography (OCT) represents one of the most reliable methods for the evaluation of morphological changes in retinal and optic nerve structure [22]. OCT changes can indicate the retrograde trans-synaptic neuronal degeneration (RTSD) of the retinal ganglion cells (RGCs) [23], being useful for the evaluation of the neurodegenerative process associated with various neurological diseases, such as multiple sclerosis [24], Alzheimer’s disease [25], Parkinson’s disease [26], and various type of headache, including migraine [23]. OCT provides information about the axonal and neuronal loss in the retina, and consequently, offers information in the visual afferent pathways and central nervous system, facilitating the diagnosis and the management of neuro-ophthalmological diseases [27]. Spectral domain OCT technology (SD-OCT) provides an individual assessment of retinal layers. SD-OCT now replaces the previous technology represented by the time domain OCT (TD-OCT). SD-OCT can give information about the peripapillary retinal nerve fiber layer (RNFL), macular ganglion cell layer, macular volume, and optic nerve [28]. The SD-OCT can assess the irreversible neuronal loss in vivo, adding a new perspective to approaching the pathophysiological mechanism associated with neuro-ophthalmological conditions, including migraine. Patients with neurodegenerative diseases have a significant decrease in the peripapillary RNFL thickness, as reported by Kwon et al. [29]. Since macula consists mainly of retinal neuron bodies and glial cells, the neuronal loss can also be determined by the assessment of the volume of the macula or macula thickness on OCT [30]. OCT is correlated with perimetry, which is the visual field test examination that can detect dysfunction in central and peripheral vision that may be caused by various medical conditions, such as ocular or neurological conditions. As compared to perimetry, OCT is more precise, highly sensitive, and easier to use in the follow-up of the patients to assess the progression of the diseases [28]. Optical coherence tomography uses interferometry to interpret reflectance data and measure RNFL thickness [10]. Macular thickness is defined as the mean thickness within the central 1000-μm diameter area [10]. The changes of RNFL thickness in migraine patients proved to be associated with the history of disease onset (number of years), the severity of disease (Migraine Disability Assessment Score (MIDAS)), the involvement of different retinal quadrants or chronic migraine onset [9,31,32,33,34]. This study aimed to assess peripapillary RNFL thickness and macula thickness by OCT examination, oxidative stress parameters, and the relationship between oxidative stress and OCT results in migraine without aura patients and with no prophylactic medication.

## 2. Materials and Methods 

A prospective, comparative, and analytical study was performed on a migraine group as compared to an age-matched group of healthy volunteers. The study protocol was approved by the Institutional Ethics Committee of the Rehabilitation Hospital, Cluj-Napoca, Romania (IECGH 4/2018). All participants included in the study signed prior to clinical examination, blood sampling, and OCT examination the written informed consent. The patients were recruited from the Neurology Clinic of Rehabilitation Hospital, Cluj-Napoca, Romania, from January to December 2018. The controls were recruited from the same hospital in the same period of time among the subjects that call for a visual check after the exclusion of those who recall migraine attacks. The Declaration of Helsinki specifications were followed. All the participants signed informed consent for participation in this study. The assessment was made in the first 24 h after the migraine attack onset.

The neurological and ophthalmological examination was done to all subjects included in the study. In the neurological examination, all the patients in the study group were selected following diagnostic criteria established by the International Headache Society (ICHD-2) [35]. Migraine severity was assessed by the neurologist using a Migraine Disability Assessment Score (MIDAS) questionnaire [36]. MIDAS score was divided into four grades: grade I (*infrequent disability*, score from 0 to 5), grade II (*mild disability*, score from 6 to 10), grade III (*moderate disability*, score from 11 to 20), and grade IV (*severe disability*, score >20). The duration of disease expressed in years and the number of attacks per month were established by anamnesis. After the neurological examination, patients with epilepsy, cerebrovascular or cardiovascular diseases, neurodegenerative diseases, hypertension, or diabetes mellitus were excluded. Patients have undergone a complete ophthalmological examination at the Ophthalmological Department of the Rehabilitation Hospital. The ophthalmologic examination included best-corrected visual acuity tested with Snellen chart, slit lamp biomicroscopy, dilatated fundus examination, intraocular pressure measurement, and visual field examination by automated perimeter (Humphrey Visual field Analyzer 750i, Zeiss, Germany). Ophthalmological examination excluded patients with glaucoma, optic nerve diseases, retinal vascular diseases, pre-proliferative or proliferative diabetic retinopathy, macular degeneration, hereditary retinal dystrophy. None of the patients with migraine had prophylactic medication prior to the presentation, and they only used triptans for migraine attacks. Other exclusion criteria included obesity, oral contraceptive medication, glucocorticoid medication, and anti-oxidant agents’ treatments.

### 2.1. Retinal Fiber Layer Thickness Assessment by Optical Coherence Tomography 

All patients and controls who met the neurological and ophthalmological criteria have been examined with ocular coherence tomography spectral domain (OCT-SD) (DRI Triton, Topcon, Tokyo, Japan) after pupillary dilatation, for both eyes. The software automatically recognizes eight retina layers. Peripapillary retinal fiber layer thickness (RNFL) and macular thickness (M) were assessed. Using macular thickness analysis mode and through a software algorithm, the device automatically constructed a topographic surface map (central field—1 mm centered on the fovea, inner ring bounded by 1 to 3 mm concentric circles, and outer ring bounded by 3 to 6 mm concentric circles). All fields were examined for both eyes: peripapillary RNFL thickness (superior, temporal, inferior and nasal quadrant), foveal thickness, inner macular thickness (superior, temporal, inferior, and nasal quadrant), and outer macular thickness (superior, temporal, inferior and nasal quadrant). The average macular thickness was manually determined [37].

### 2.2. Oxidative Stress Parameters

Blood samples were taken from all the subjects included in the study, in the morning, after fasting for 12 h, in the first 24 h after admission into the hospital for a migraine attack. The samples were assessed regarding the oxidative status and antioxidant capacity of plasma. The oxidative status evaluation consisted of the determination of total oxidative stress (TOS) using the method of Erel et al. [38], malonyl dialdehyde (MDA) using the method of Ohkawa et al. [39] and nitric oxide (NOx) using the method of Giustarini et al. [40]. Total NOx levels were used as a surrogate marker for serum nitric oxide levels [40]. The antioxidant capacity of plasma was assessed by measuring the level of catalase [41], and total anti-oxidative (TOS) capacity [42]. Spectroscopic measurements assessed the oxidative stress parameters. A Jasco V-350 UV-VIS spectrophotometer (Jasco International Co., Ltd., Tokyo, Japan) was used for all measurements. All the chemical substances were purchased from Sigma-Aldrich.

### 2.3. Statistical Analysis

Statistical analysis was done with Statistica program (v. 8, StatSoft, Tulsa, OK, USA), and p-values less than 0.05 were considered statistically significant. The numerical data were assessed for normal distribution and were summarized as mean (standard deviation) if normally distributed, respectively, and median (Q1 to Q3) (where Q is the quartile) otherwise. An independent *t*-test or Mann–Whitney test was used to evaluate differences between groups according to data distribution. The qualitative data were reported as a number, percentage and 95% confidence intervals (values provided in squared brackets) calculated with an exact method [43] and analyzed by the Chi-square test. For correlation analysis, Pearson’s or Spearman’s methods were used according to the distribution of data.

## 3. Results

Seventy-seven patients, forty-one with migraine and thirty-six controls, were investigated. The investigated sample comprise subjects aged from 22 to 47 years old, without significant differences between groups (median (IQR) migraine vs. control group = 39 (35–41) vs. 35.5 (29–42), Z-stat (Mann–Whitney test) = 1.3, *p* = 0.1878).

Twenty-nine (70.7%) women were included in the group with migraine and twenty-three (63.9%) in the control group, without significant differences between groups (*χ*^2^ = 0.4, *p* = 0.5223).

The time in years from the migraine diagnostic varied from 3 to 8 years with a median of 5 years (IQR = (4–6) in the group with migraine. The frequency of migraine attacks per month varied from 2 to 8, with a median of 4 (3–6). 

A different pattern was observed according to genders regarding the MIDAS score, with significantly higher frequency of MIDAS I and II score among men, and MIDAS III and IV score among women (Fisher exact test; *p* = 0.0006).

A different pattern of oxidative stress parameters was observed between groups with significantly higher values of NOx, MDA, and TOS, and, respectively, significantly lower values of catalase and TAC among the migraine group as compared to the control group (Table 1). No significant differences regarding the investigated oxidative stress parameters were observed between genders, neither in the migraine group (Mann–Whitney test, *p* > 0.18) nor in the control group (Mann–Whitney test, *p* > 0.31).

The thickness of the peripapillary retinal nerve fibers proved to be, without any exceptions, significantly smaller in the migraine group as compared to the control group (Table 2).

The averages of macular RNFL thickness for the outer and inner ring were significantly reduced in patients with migraine compared with the control group with a few exceptions: an inferior quadrant of the outer ring of the fovea for the left eye, an inferior quadrant of the inner ring and a superior quadrant of the outer ring of the fovea for the left eye (Table 3).

The most frequent significant relationships were observed with catalase, and the most involved quadrant in these correlations was the temporal quadrant that showed a strong positive correlation with macular RNFL thickness, inner ring in both eyes (Table 4). The strongest positive correlation was also found for catalase, and peripapillary RNFL thickness nasal quadrant left eye (*p* = 0.007) (Table 4).

## 4. Discussion

Our study identified increased values of the oxidative stress parameters (NOx, MDA, and TOS), decreased values of both parameters for anti-oxidative status, decreased peripapillary retinal nerve fiber layer thickness as well as macular RNFL thickness among those with migraine as compared to those without migraine. Furthermore, several significant correlations were identified between oxidative stress parameters and, respectively, catalase on the one hand and peripapillary and macular thickness, on the other hand.

A higher percentage of women with migraine was observed in our study and this result is expected since female sex is considered a risk factor for migraine onset as well as for its transformation into a chronic disorder [44]. The prevalence of migraine in the general population is reported as three-times more in women than in men, especially during the reproductive years [45], our results being closed to this ratio. Furthermore, a more significant disability associated with female gender is reported [46], this also being observed in our study with a higher disability according to MIDAS score among women as compared to men. The role of estrogens in migraine risk and clinical characteristics, with decreasing levels in the premenstrual period associated with menstrual-related migraine, had already been demonstrated [45]. Gender is also associated with differences in oxidative stress, women appear to be less susceptible to oxidative stress due to the antioxidant properties of the estrogens [47,48].

### 4.1. Migraine and Oxidant/Antioxidant Balance Assessment

Oxidative stress is a plausible unifying principle behind the types of migraine triggers encountered in clinical practice [49]. All of the parameters that reflect the oxidant/anti-oxidant balance in our study (NOx, MDA, TOS, catalase, and TAC) were found to be significantly modified in migraine patients compared with the control group (Table 1). Endogenous reactive species such as reactive oxygen species (ROS) and reactive nitrogen species (RNS) are emerging as molecules that can mediate cell signaling responses with the activation of Ca^2+^-permeable membrane channels, encoded by the transient receptor potential (TRP) gene superfamily that are characterized by a wide variety of activation triggers that act from outside and inside on the cells [50]. Members of one class of TRP channels have emerged as sensors not only for reactive oxygen species (ROS), or reactive nitrogen species (RNS) but also for reactive carbonyl species (RCS), and gaseous messenger molecules including molecular oxygen (O_2_), hydrogen sulfide (H_2_S), and carbon dioxide (CO_2_) [51]. Transient receptor potential ankyrin-1 (TRPA1) ion channels, found on pain-sensitive nerve endings in the dura mater, play the role of nociceptor and reacts to environmental oxidative stress, transducing it into a neural signal, resulting in pain perception [50]. An associated neurogenic inflammation will also result, and will amplify the nociception process [52] reflected by pain-related disability (MIDAS score). Endogenous ROS can cause damage to DNA, mitochondria, membrane lipids, and proteins and, consequently, can contribute to neuronal cell loss in migraine patients [48,53,54] that were assessed in our study by OCT examination (Table 2 and Table 3). There is a selective sensitivity of neurons in the central nervous system to oxidative stress [53], some of these regions being involved in the pathophysiology of a migraine attack (for example, functional connectivity between the hypothalamus and brainstem was found to be altered during a migraine attack, leading to the hypothesis that this network change might be the real driver of migraine attacks [55]). According to these data, we can also hypothesize that the vulnerability of specific neuronal networks to oxidative stress can initiate and maintain the migraine attacks. However, the oxidative stress during migraine attacks may constitute a physiologic environment conducive to stem cells [56]. Still, migraine attack is followed by the neuronal loss [57] and, as a consequence, the neuroplasticity phenomena can initiate an integrated mechanism for neural repair [49]. The balance between neuronal loss during migraine attack and neuronal repair stimulation after migraine attack has to be controlled in order to be used as a beneficial phenomenon for using it as a therapy for facilitating the survival, proliferation, migration, and differentiation of stem cells according to Borkum theory [49].

One of the most important molecules for reactive species is nitric oxide, the smallest signaling molecule known, that will enhance nitro-oxidative stress due to its increased production. Increasing NO production can be a result of the activation of three isoforms of NO synthase (NOS): neuronal NOS (nNOS), inducible NOS (iNOS), and endothelial NOS (eNOS) [57]. Their activation can exert various effects: nNOS activation contributes to synaptic plasticity in the central nervous system (CNS), central regulation of blood pressure, smooth muscle relaxation, and vasodilatation, iNOs activation contributes to the pathophysiology of inflammation, and eNOS activation has an important role for vasodilatation and vasoprotective effect [58]. Our results demonstrate a significantly increased NO molecule in migraine patients compared with control subjects (Table 1). Increased NO production can result from the activation of all of the three NOS isoforms. The NO excess reacts with superoxide, leading to the creation of peroxynitrite, a damaging product [58,59]. Several isoforms of O_2_^•−^-producing NADPH oxidase exists in the vascular wall [60]. Many types of vascular diseases appear as being associated with the up-regulation of NADPH oxidase [57] and evidence suggested that migraine pathophysiology is connected with vascular reactivity [61,62]. Endothelial NOS (eNOS) appears mainly involved in migraine pathophysiology. NO produced by eNOS that has been activated by serotonin (released by activated platelets), contributes to the vasodilation of migraines and associated neurogenic inflammation by stimulating the release of substance P that is an important molecule involved in neurogenic inflammation [63,64]. eNOS is also involved in endothelium, releasing VEGF and BDNF that are implicated in central sensitization during migraine attacks [65].

Among the constituents that contribute to increased oxidative stress in migraine patients, compared with the control group, malondialdehyde (MDA) is the constituent that features in our study (Table 1). A significantly increased MDA reflects an intense lipid peroxidation as an important contributor to oxidative stress and neuronal loss since neuronal membranes are rich in polyunsaturated fatty acids and are particularly susceptible to oxidative stress [66]. TOS was significantly increased and TAS was found to be significantly decreased in migraine patients compared to controls by our study, a result similar to the Yigit et al. [67] but opposite to the study conducted by Geyik et al. who reported no significant difference [68]. The age of the investigated patients can explain our different results regarding the oxidative stress changes as compared to Geyik et al. for our migraine group [68] aging itself being an essential contributor to increased oxidative stress [69]. Similar to our findings, Alp et al. reported significantly decreased TAC in migraine patients [70]. Several studies also reported that different types of headache are associated with decreased antioxidant defenses mechanisms [71,72,73]. As in our study (Table 1), the catalase level, as a contributor to the antioxidant system, was reported to be significantly decreased in migraine patients [74,75]. The persistence of oxidative stress and increasing the frequency of migraine attacks can lead to a chronic migraine [2]. Adding all of these pieces of evidence to our study, regarding the oxidative stress in migraine patients, we are considering that the disturbances of oxidant/antioxidant system balance has an important contribution in migraine pathogenesis. 

### 4.2. Migraine and RNFL Thickness Assessment 

The retinal nerve fiber layer (RNFL) contains the axons of the retinal ganglion cells. Therefore, measurement of the RNFL thickness by OCT is expected to provide information for monitoring the progressive loss of ganglion cells and axons in migraine patients [23].

Our study demonstrated that retinal fiber layers are significantly decreased in patients with migraine (peripapillary RNFL thickness for migraine group was decreased for all quadrants) (Table 2). For the macular region, fovea RNFL thickness was significantly decreased for both eyes (Table 3). Our hypothesis is that oxidative stress contributes to RFLN thickness changes, and can be an effect of each recurrent attack since there are significant differences between the control group and migraine patients. Despite the inconsistent significant modification of macular thickness for various quadrants, macular volume significantly decreased in both eyes in migraine patients compared with the control group (Table 2 and Table 3). Previous OCT studies had also demonstrated a significant decrease in RNFL thickness in various quadrants in patients with migraine. Assessing migraine patients by TD-OCT (time domain optical coherence tomography), different results were reported. Reduced RNFL thickness in the nasal sector was found by Sorkhabo et al. in a case-control study [34]. Martinez et al. found thinner TD-OCT RNFL in the temporal sector in migraine patients compared with healthy volunteers [76]. Colak et al. found on migraine patients using SD-OCT (spectral domain optical coherence tomography) no significant difference in the RNFL thicknesses of the temporal and nasal quadrants compared with control subjects but a significant difference of the superior and inferior quadrants [77]. In the same study, foveal, temporal, and nasal choroidal thickness measurements were significantly lower in the migraine group than in the control group [76]. Decreased thickness of RNFL in nasal sectors in patients with migraine (assessed by SD-OCT) was also found by different studies [78,79].

### 4.3. Correlation Between Oxidant/Antioxidant Balance and RNFL Thickness

Oxidative stress may be a final common pathway, signaling several unfavorable conditions in the brain, including neuronal degeneration and apoptosis [58]. Studies regarding retinal neuronal fiber layer involvement in migraine patients revealed a possible connection between the thickness of RNFL and migraine pathophysiology, even if different studies report discrepancies in the involved quadrants. Our results show a statistically significant correlation between the reduction in RNFL in the peripapillary region in different quadrants with different oxidative stress/anti-oxidative parameters with the strongest positive significant correlation (*p* < 0.007) between peripapillary thickness in nasal quadrants and catalase, in migraine patients (Table 4). Furthermore, a significant correlation for all quadrants with different stress oxidative parameters were also identified most often, the temporal quadrant being correlated for peripapillary and macular thickness with MDA, TOS, and catalase (Table 4). Our results suggest the absence of an identifiable rule regarding the involved quadrants of RNFL thickness and various oxidative parameters tested. However, the results obtained on this pilot study must be first validated on a larger sample, and if are reproducing, the mechanisms must be assessed. Reported results regarding the correlation of RNFL thickness with various parameters also showed associations that are not following a precise pattern. Abdellatif et al. [80] found that the duration of migraine is significantly correlated with the thickness of the ganglion cell layer, retinal nerve fiber layer, and all choroidal quadrants. The severity of migraine was only significantly correlated with the thickness of the ganglion cell layer and the retinal nerve fiber layer [79]. The duration of a migraine attack is the most important determinant factor for the decrease in the thickness of the superior retinal nerve fiber and in all the choroidal quadrants, while the severity of migraine is the most important determinant factor of inferior, nasal, and temporal retinal nerve fiber layer quadrants and the inferior ganglion cell layer [79]. Also using a spectral-domain optical coherence tomography assessment, some researchers found that inferior and superior quadrant RNFL thickness was significantly thinner in patients with migraine compared with the control [81]. Comparing both eyes in migraine patients with control subjects, the nasal RNFL thicknesses were significantly thinner on the right side in our study, while Gunes et al. reported a similar result but on both sides [82]. Other different significant correlations between RNFL thickness and migraine characteristics were also reported: the length of the history of a migraine [21], the migraine disability score [34], or visual disturbances amplitudes [83].

Consequently, we believe that the OCT-based evaluation of RNFL thickness could offer a non-invasive method for evaluating axonal loss in migraine patients. From our knowledge, this is the first study investigating the association between the oxidative stress parameters and RNFL thickness, a mechanism that could be an essential step for migraine treatment, targeting oxidative stress molecules. In light of this fact, personalized therapy could be applied according to which specific oxidative stress/antioxidant molecule is increased in each patient.

### 4.4. Study Limitation and Perspectives

Several limitations to our study could be listed. First, the presented study was designed as a pilot study on a small sample of patients having migraines without aura. The observed pattern of association between RNFL thickness in different quadrants and various oxidative stress parameters must be validated on larger sample sizes. Furthermore, the assessment of these associations among men and women on a larger sample size could be of scientific interest. Second, we evaluated patients with migraine with no prophylactic medication, but insufficient data were collected regarding the prophylaxis (such as were the medications never used or interrupted, were they interrupted recently or since a prolonged interruption, which medications were used, what doses were given and for how long, etc.). Third, the assessment of the group with migraine was made in the first 24 h after the migraine attack onset. Migraine attacks are known to be accompanied by oedema of the forearm, eyelids, and cheeks. The evaluation of the retinal parameters between attacks, out of painful periods, could bring more insights into how these parameters change during attacks and free of symptoms periods, allowing for a better assessment of the disease evolution. Fourth, we only evaluated the persons with migraines without aura. An evaluation also conducted on patients with migraine with aura could identify the same pattern or a different pattern of changes and associations between RNFL thickness and various oxidative stress parameters. Fifth, the results reported in correlation analysis must be interpreted with caution, because some statistically significant correlation could be observed, by chance, considering the number of the ocular coherence tomography spectral measurements.

## 5. Conclusions

Decreasing the RNFL thickness in migraine patients, in different quadrants, can be associated with an imbalance between oxidative stress and antioxidants by increasing oxidative stress or by reducing the antioxidant mechanisms, contributing to the axon degeneration in retinal layers. Because retina contains axons associated with ganglion cell neurons, and the RNFL is abundant in axonal tissue and has no myelin, the OCT assessment of RNFL thickness can be a potentially useful biomarker of axonal loss in the central nervous system. The association of RNFL thickness with various oxidative stress parameters in migraine patients can constitute an essential step for personalized therapy focused on targeting reducing oxidative stress molecules. However, the role of oxidative stress in migraine pathophysiology is not yet completely and worldwide established. This is a pilot study, and more exhaustive studies are needed to produce evidence for clinical practice.

## Figures and Tables

**Table 1:** Oxidative stress parameters by groups.

	Migraine Group (*n* = 41)	Control Group (*n* = 36)	t-Stat. (*p*)
NOx (µmol/L)			28.3 (<0.001)
Mean (SD)	43.0 (3.1)	26.4 (1.8)	
(Min to Max)	36.0–49.0	23.0–30.0	
MDA (nmol/L)			57.8 (<0.001)
Mean (SD)	6.2 (0.3)	2.6 (0.2)	
(Min to Max)	5.8–6.9		
TOS (µmol H_2_O_2_/L)			70.6 (<0.001)
Mean (SD)	34.4 (1.6)	13.4 (0.9)	
(Min to Max)	30.0–37.0	11.4–15.1	
Catalase (U/ml)			−50.2 (<0.001)
Mean (SD)	34.5 (2.3)	68.4 (3.6)	
(Min to Max)	31.0–40.7	60.7–74.2	
TAC (mmol trolox/L)			−14.5 (<0.001)
Mean (SD)	1.0 (0.2)	1.8 (0.3)	
(Min to Max)	0.6–1.4	1.1–2.5	

**Table 2:** Peripapillary retinal nerve fiber layer thickness (µm) by groups.

	Migraine Group (*n* = 41)	Control Group (*n* = 36)	t-Stat. (*p*)
Right Eye Quadrants			
Superior	103.0 (5.9)	118.3 (5.7)	−11.5 (<0.001)
Temporal	58.0 (6.4)	80.8 (5.2)	−16.9 (<0.001)
Inferior	110.1 (5.4)	122.2 (4.1)	−10.9 (<0.001)
Nasal	78.1 (3.8)	81.1 (4.6)	−3.00 (0.003)
Left Eye Quadrants			
Superior	109.9 (7.1)	118.1 (5.3)	−5.7 (<0.001)
Temporal	59.4 (2.8)	76.9 (2.5)	−28.4 (<0.001)
Inferior	116.6 (4.6)	120.5 (4.4)	−3.8 (0.001)
Nasal *	80.0 (79.0–85.0)	85.0 (80.0–88.3)	−2.5 (0.010)

Student *t*-test was applied for normally distributed measurements (reported as mean (standard deviation)). Excepting those with * (reported as median (Q1–Q3), where Q is the quartile) when Mann-Whitney test was applied.

**Table 3:** Macular retinal nerve fiber layer (RNFL) thickness (µm) analysis by groups.

	Migraine Group (*n* = 41)	Control Group (*n* = 36)	t-Stat. (*p*)
Right eye quadrants			
Fovea	251.5 (9.9)	270.2 (6.8)	−9.6 (<0.001)
*Inner ring*			
Superior	277.9 (9.5)	283.0 (7.8)	−2.6 (0.012)
Temporal	230.0 (9.6)	283.2 (6.5)	−28.1 (<0.001)
Inferior	260.1 (7.0)	267.3 (6.1)	−4.8 (<0.001)
Nasal	282.6 (11.3)	290.9 (9.2)	−3.5 (0.001)
*Outer ring*			
Superior	236.9 (6.8)	241.7 (6.5)	−3.1 (0.003)
Temporal	216.2 (5.1)	239.3 (4.6)	−20.7 (<0.001)
Inferior	262.2 (8.1)	264.3 (9.2)	−1.0 (0.313)
Nasal	272.1 (9.1)	278.1 (8.7)	−3.0 (0.004)
Macular volume	8.3 (0.5)	8.7 (0.6)	−3.8 (<0.001)
Left eye quadrants			
Fovea	247.4 (7.9)	284.4 (11.2)	−16.9 (<0.001)
*Inner ring*			
Superior	271.0 (8.6)	279.2 (11.3)	−3.6 (0.001)
Temporal	224.0 (9.6)	278.3 (11.3)	−22.7 (<0.001)
Inferior	263.5 (9.2)	267.6 (10.0)	−1.8 (0.071)
Nasal	281.1 (10.8)	289.3 (11.8)	−3.2 (0.002)
*Outer ring*			
Superior	244.0 (8.5)	247.2 (8.8)	−1.7 (0.102)
Temporal	218.2 (13.0)	242.6 (9.1)	−9.4 (<0.001)
Inferior	259.2 (6.7)	263.3 (10.2)	−2.1 (0.039)
Nasal	267.1 (10.4)	273.0 (11.5)	−2.4 (0.021)
Macular volume	7.8 (0.6)	8.7 (0.4)	−7.4 (<0.001)

**Table 4:** Pearson’s rank correlations (r) among patients with migraine.

Characteristics	r (*p*)
Peripapillary thickness (µm)	
MDA & Peripapillary RNFL thickness-S-LE	0.31 (0.048)
TOS & Peripapillary RNFL thickness-T-LE	0.41 (0.009)
Catalase & Peripapillary RNFL thickness-N-LE	0.41 (0.007)
TAC & Peripapillary RNFL thickness-S-LE	−0.34 (0.029)
Macular thickness (µm)	
Catalase & Fovea-LE	0.31 (0.044)
NOx & Macular RNFL thickness-InnerRing-I-RE	0.39 (0.013)
MDA & Macular RNFL thickness-InnerRing-T-LE	−0.37 (0.017)
Catalase & Macular RNFL thickness-InnerRing-T-RE	−0.38 (0.014)
Catalase & Macular RNFL thickness-InnerRing-T-LE	−0.39 (0.012)
TAC & Macular RNFL thickness-InnerRing-N-LE	0.40 (0.010)
NOx & Macular RNFL thickness OuterRing-I-RE	0.34 (0.028)
TOS & Macular RNFL thickness OuterRing-S-RE	0.39 (0.011)
TAC & Macular RNFL thickness OuterRing-N-RE	−0.34 (0.031)

r = Pearson’s rank correlation coefficient, TOS = total oxidative stress, NOx = nitric oxide, TAC = total anti-oxidative capacity, MV = macular volume, MDA = malonyl dialdehyde, LE = left eye, RE = right eye, T = temporal quadrant, N = nasal quadrant, S = superior quadrant, I = inferior quadrant.
